# Concussion knowledge, attitudes and reporting intention among adult competitive Muay Thai kickboxing athletes: a cross-sectional study

**DOI:** 10.1186/s40621-018-0155-x

**Published:** 2018-06-11

**Authors:** Reidar P. Lystad, Stephen J. Strotmeyer

**Affiliations:** 1 Australian Institute of Health Innovation, Macquarie University, Level 6, 75 Talavera Road NSW, Sydney, 2109 Australia; 2 Division of General Surgery and Trauma, Department of Surgery, University of Pittsburgh Medical Center, Children’s Hospital of Pittsburgh of UPMC, Pittsburgh, Pennsylvania USA

**Keywords:** Brain concussion, Health knowledge, Attitudes, Practice, Combat sport, Kickboxing

## Abstract

**Background:**

Muay Thai kickboxing is a full-contact combat sport with a high incidence of head trauma, yet little is known about these athletes’ concussion knowledge and attitudes. This study aims to describe and quantify concussion knowledge, attitudes, and reporting intention among adult competitive Muay Thai kickboxing athletes.

**Results:**

This cross-sectional study comprised 193 competitive Muay Thai kickboxing athletes aged ≥18 years registered with the Thai Boxing Association Sanctioning Authority. The mean concussion knowledge score was 19.5 (*SD* 2.3) out of 25, the mean concussion attitudes score was 62.7 (*SD* 7.4) out of 75, and 134 (69.4%) of respondents indicated that they were likely to report concussion symptoms. No significant predictors of concussion knowledge, attitudes, or reporting intention were revealed.

**Conclusions:**

Competitive Muay Thai kickboxing athletes appear to have reasonably good concussion knowledge, attitudes, and reporting intention; however, there is still room for further improvement. It is recommended that concussion education programs based on current best evidence are made available to all kickboxing athletes and coaches.

## Background

Concussion is recognized as a major and increasing public health issue, with sport and active recreation being a common cause (Finch et al., 2013; Pfister et al., 2016). The Centers for Disease Control and Prevention estimating that around 3 million concussions occur in sports and recreational activities each year in the United States, with a cumulative cost of US$56 billion (Langlois et al., 2006). However, the true incidence of concussion may be far greater because sports-related concussion is often unrecognized and unreported by athletes and coaches and undiagnosed by doctors (Delaney et al., 2002; Delaney et al., 2015). Unrecognized concussions are problematic because the associated transient decline in neurocognitive function and premature return to sport substantially increases the risk of subsequent injury (Brooks et al., 2016; Herman et al., 2017), including serious injury such as second impact syndrome (Guskiewicz et al., 2003; McCrory et al., 2017).

Concussion has become an increasingly prevalent topic in both the scientific literature and the popular media (Baker et al., 2017; McCrory et al., 2017). The number of Google internet searches for concussion has markedly increased worldwide since in 2009 (Lawson McLean et al., 2016), which suggests an increasing public interest in concussion. In the United States, there has been a large increase in the number of people seeking healthcare for concussion, which has been attributed to an increase in the general public awareness of concussion (Baker et al., 2017). It is less clear, however, whether the increased awareness in the general public is mirrored by increased knowledge about concussion symptoms and appropriate behavior among athletes.

Prior to the elevated public awareness one study reported that only 23% of concussed football players and 20% of concussed soccer players realized that they had suffered a concussion (Delaney et al., 2002), while a study published in 2014 found that 50% of concussed ice hockey players knew they had sustained a concussion (Kroshus et al., 2014). To make matters worse, superior concussion knowledge does not necessarily translate to appropriate concussion reporting behaviour (Kroshus et al., 2015; Kroshus et al., 2014; Register-Mihalik et al., 2013). Motivational factors are often key to engage in appropriate behavior, and reporting intention has been shown to be a valuable metric for predicting concussion reporting behaviour (Kroshus et al., 2015).

Full-contact combat sport athletes have a high risk of injury, including head trauma (Lystad, 2015a). Concussion rates are high in full-contact combat sports that emphasize delivering blows to the head (e.g., boxing, kickboxing, and mixed martial arts) (Lystad, 2015a); however, there is good reason to believe that substantial underreporting occurs (Heath & Callahan, 2013; Hutchison et al., 2014; Lystad, 2015b). Despite the high incidence of concussion in full-contact combat sports, little is known about the attitudes towards and knowledge of concussion among these athletes. This lack of information makes it difficult to evaluate whether interventions such as concussion education programs could be potentially beneficial in mitigating negative consequences of concussion in combat sport athletes. The purpose of this study, therefore, was to describe and quantify concussion attitudes, knowledge, and reporting-intention among competitive Muay Thai kickboxing athletes in North America.

## Methods

### Participants

Adult athletes registered in the Thai Boxing Association Sanctioning Authority (TBA-SA) database were invited to participate in this study. The TBA-SA is the largest body to promote and sanction amateur Muay Thai kickboxing events in North America. The TBA-SA mailing list comprised 1,450 contact email addresses, of which 1,309 belonged to athletes aged ≥18 years. Of these, 743 were considered to be recently active athletes (i.e. they had participated in at least one contest in the last two years).

### Procedures

Potential participants received three invitations to participate in this study via email, one initial invitation and two follow-up emails over the subsequent two weeks. Eligible participants were asked to complete an anonymous online survey administered via Qualtrics research software platform (Qualtrics, Provo, UT, USA). The online survey comprised two parts: demographics and the Rosenbaum Concussion Knowledge and Attitudes Survey – Student Version (RoCKAS-ST) (Rosenbaum & Arnett, 2010).

### Outcome measures

Concussion knowledge and attitudes were measured using the previously validated RoCKAS-ST (Rosenbaum & Arnett, 2010; Chapman et al., 2018). The instrument comprises 55 items, from which Concussion Knowledge Index (CKI) and Concussion Attitude Index (CAI) composite scores are derived. The possible range of CKI scores is from 0–25, with higher scores indicating higher levels of concussion knowledge. The possible range of CAI scores is from 15–75, where higher scores represent safer attitudes about concussion. Concussion symptom reporting intention was measured using a single item from Section 3 of the RoCKAS-ST. The item states “I would continue playing a sport while also having a headache that resulted from a minor concussion” and is scored on a 5-point Likert scale, with higher scores indicating stronger agreement with the statement. As per Kroshus and colleagues (Kroshus et al., 2015), this item was considered to be a face valid proxy for symptom reporting intention as it reflects an athlete’s overall appraisal of their likelihood of engaging in the opposite behavior of symptom reporting (i.e. continued play while symptomatic). Thus, disagreement with the statement was considered the “correct” response (i.e. likely to report concussion symptoms), and vice versa.

### Data analysis

All analyses were conducted using R version 3.4.2 (R Foundation for Statistical Computing, Vienna, Austria). Descriptive statistics were used to summarise demographic characteristics (i.e. sex, age, level of experience, and concussion history) and outcome measures (i.e. concussion knowledge, attitudes, and reporting intention). Separate multivariable linear regression models were constructed for concussion knowledge and attitudes, and a multivariable ordinal regression model was fitted for concussion reporting intention. The independent variables relating to level of experience (i.e. number of years training and number of fights contested) were log-transformed prior to fitting regression models. An alpha level of 0.05 was used as threshold for statistical significance.

### Ethical considerations

This study was approved by the University of Pittsburgh Institutional Review Board (reference number: PRO16100336).

## Results

A total of 193 eligible respondents completed the online survey, for an estimated response rate of 26.0% (193/742). The characteristics of the sample are shown in Table 1. More than two thirds (67.7%) of the respondents were male and the mean age was 31.3 (*SD* 7.6) years. In regard to level of experience, the respondents had, on average, trained for 6.5 (*SD* 4.4) years and participated in 4.1 (*SD* 3.7) fights. More than half of the respondents (58.0%) had a history of suspected concussion (median = 2), while only 20% reported having ever been diagnosed with concussion.

**Table 1:** Characteristics of the study sample (*N* = 193)

Characteristic	Frequency (%)
Sex	
Male	123 (67.7%)
Female	70 (36.3%)
Age	
18–24 years	33 (17.1%)
25–34 years	107 (55.4%)
35–44 years	46 (23.8%)
≥45 years	7 (3.6%)
Level	
Amateur	179 (92.7%)
Professional	14 (7.3%)
Training experience	
0–2 years	20 (10.4%)
3–5 years	74 (38.3%)
6–9 years	63 (32.6%)
≥10 years	30 (15.5%)
Fight experience	
0–2 fights	37 (19.2%)
3–5 fights	52 (26.9%)
6–9 fights	35 (18.1%)
≥10 fights	59 (30.6%)
History of diagnosed concussion	
Yes	38 (19.7%)
No	155 (80.3%)
History of suspected concussion^a^	
Yes	112 (58.0%)
No	80 (41.5%)

^a^One response missing

Overall, the mean CKI and CAI scores were 19.5 (*SD* 2.3) and 62.7 (*SD* 7.4), respectively. Table 2 shows the mean CKI and CAI scores stratified by age group, sex, level of experience, and concussion history. In regard to reporting intention, 134 (69.4%) of respondents indicated that they were likely to report concussion symptoms (i.e. “correct” response), while 33 (17.1%) of respondents indicated that they were unlikely to report concussion symptoms (i.e. “incorrect” response). Table 3 shows the concussion reporting intention responses stratified by age group, sex, level of experience, and concussion history.

**Table 2:** Mean concussion knowledge and attitudes scores with standard deviation, stratified by sample characteristic

Characteristic	Mean CKI score (*SD*)	Mean CAI score (*SD*)
Sex		
Male	19.6 (2.4)	62.6 (8.1)
Female	20.0 (1.8	63.8 (6.3)
Age		
18–24 years	19.4 (2.4)	60.9 (8.3)
25–34 years	19.8 (2.2)	63.0 (7.3)
35–44 years	20.1 (1.9)	64.4 (7.4)
≥45 years	18.0 (2.7)	65.1 (6.2)
Level		
Amateur	19.8 (2.2)	63.0 (7.7)
Professional	19.1 (2.8)	64.0 (5.0)
Training experience		
0–2 years	19.5 (2.5)	63.0 (9.7)
3–5 years	19.4 (2.5)	61.7 (7.6)
6–9 years	19.9 (2.1)	63.7 (6.8)
≥10 years	20.1 (1.6)	64.3 (7.1)
Fight experience		
0–2 fights	19.8 (2.6)	62.1 (9.4)
3–5 fights	19.8 (2.3)	63.9 (7.5)
6–9 fights	19.6 (2.2)	62.4 (6.7)
≥10 fights	19.8 (2.0)	63.3 (7.2)
History of diagnosed concussion		
Yes	20.2 (2.0)	65.0 (5.8)
No	19.6 (2.3)	62.6 (7.8)
History of suspected concussion^a^		
Yes	19.9 (2.1)	63.2 (7.5)
No	19.5 (2.4)	62.7 (7.6)

^a^One response missing

*CAI* Concussion Attitudes Index, *CKI* Concussion Knowledge Index

**Table 3:** Frequency and proportion of concussion reporting intention responses, stratified by sample characteristic

Characteristic	Frequency (%)		
	Correct	Neutral	Incorrect
Sex			
Male	86 (69.9%)	17 (13.8%)	20 (16.3%)
Female	48 (68.6%)	9 (12.9%)	13 (18.6%)
Age			
18–24 years	19 (57.6%)	9 (27.3%)	5 (15.2%)
25–34 years	73 (68.2%)	17 (15.9%)	17 (15.9%)
35–44 years	36 (78.3%)	0 (0%)	10 (21.7%)
≥45 years	6 (85.7%)	0 (0%)	1 (14.3%)
Level			
Amateur	125 (69.8%)	24 (13.4%)	30 (16.8%)
Professional	9 (64.3%)	2 (14.3%)	3 (21.4%)
Training experience			
0–2 years	10 (50%)	5 (25%)	5 (25%)
3–5 years	47 (63.5%)	15 (20.3%)	12 (16.2%)
6–9 years	49 (77.8%)	5 (7.9%)	9 (14.3%)
≥10 years	22 (73.3%)	1 (3.3%)	7 (23.3%)
Fight experience			
0–2 fights	24 (64.9%)	3 (13.5%)	8 (21.6%)
3–5 fights	37 (71.2%)	9 (17.3%)	6 (11.5%)
6–9 fights	25 (71.4%)	5 (14.3%)	5 (14.3%)
≥10 fights	42 (71.2%)	7 (11.9%)	10 (16.9%)
History of diagnosed concussion			
Yes	27 (71.1%)	2 (5.3%)	9 (23.7%)
No	107 (69.0%)	24 (15.5%)	24 (15.5%)
History of suspected concussion^a^			
Yes	81 (72.3%)	13 (11.6%)	18 (16.1%)
No	52 (65.0%)	13 (16.3%)	15 (18.8%)

^a^One response missing

The regression models for concussion knowledge and concussion attitudes displayed no evidence of explanatory variables collectively predicting CKI scores (*F*_7,183_ = 1.39; *P* = 0.21) or CAI scores (*F*_7,183_ = 1.35; *P* = 0.23), respectively. The models explained only 5% of the variability in CKI scores (*R*^*2*^ = 0.05) and CAI scores (*R*^*2*^ = 0.05). The ordinal logistic regression model did not reveal any significant differences in odds for any of the explanatory variables and concussion reporting intention (Table 4).

**Table 4:** Ordinal logistic regression of the relationship between concussion reporting intention and explanatory variables

Explanatory variable	OR	95%CI	*P* value
Sex (ref. Male)	1.06	0.62–1.83	0.825
Age, years	0.97	0.93–1.01	0.144
Level (ref. Amateur)	1.35	0.44–4.00	0.590
Training experience, log no. years	0.59	0.28–1.21	0.150
Fight experience, log no. fights	1.09	0.74–1.62	0.657
History of diagnosed concussion (ref. Yes)	0.71	0.36–1.40	0.325
History of suspected concussion (ref. Yes)	1.10	0.63–1.92	0.748

*OR* odds ratio, *CI* confidence interval

Table 5 provides an overview of the frequency and proportions of correct responses to the CKI items. Although the majority of concussion knowledge items were correctly answered by a high proportion of respondents, a few items were noticeable for their very low proportion of correct responses. For instance, only 19.7% of respondents correctly distinguished loss of consciousness from coma, and only 30.6% of respondents correctly stated that brain imaging typically shows no visible physical damage after concussion.

**Table 5:** Frequency and proportion of correct responses to concussion knowledge index items

Concussion Knowledge Index (CKI) item	Frequency (*%*)
Section 1 (Statements – True / False)	
There is a possible risk of death if a second concussion occurs before the first one has healed.	175 (90.7%)
People who have had one concussion are more likely to have another concussion.	173 (89.6%)
In order to be diagnosed with a concussion, you have to be knocked out.	191 (99.0%)
A concussion can only occur if there is a direct hit to the head.	157 (81.3%)
Being knocked unconscious always causes permanent damage to the brain.	132 (68.4%)
Symptoms of a concussion can last for several weeks.	190 (98.4%)
Sometimes a second concussion can help a person remember things that were forgotten after the first concussion.	172 (89.1%)
After a concussion occurs, brain imaging (e.g., CAT Scan, MRI, X-Ray, etc.) typically shows visible physical damage (e.g., bruise, blood clot) to the brain.	59 (30.6%)
If you receive one concussion and you have never had a concussion before, you will become less intelligent.	186 (96.4%)
After 10 days, symptoms of a concussion are usually completely gone.	79 (40.9%)
After a concussion, people can forget who they are and not recognize others but be perfect in every other way.	55 (28.5%)
Concussions can sometimes lead to emotional disruptions.	187 (96.9%)
An athlete who gets knocked out after getting a concussion is experiencing a coma.	38 (19.7%)
There is rarely a risk to long-term health and well-being from multiple concussions.	171 (88.6%)
Section 2 (Scenarios – True / False)	
*Scenario 1:* *While playing in a game, Player Q and Player X collide with each other and each suffers a concussion. Player Q has never had a concussion in the past. Player X has had 4 concussions in the past.*	
It is likely that Player Q’s concussion will affect his long-term health and well-being.	121 (62.7%)
It is likely that Player X’s concussion will affect his long-term health and well-being.	177 (91.7%)
*Scenario 2:* *Player F suffered a concussion in a game. She continued to play in the same game despite the fact that she continued to feel the effects of the concussion.*	
Even though Player F is still experiencing the effects of the concussion, her performance will be the same as it would be had she not suffered a concussion.	175 (90.7%)
Section 5 (Symptom recognition – Check all that apply)	
Headache	184 (95.3%)
Sensitivity to light	171 (88.6%)
Difficulty remembering	173 (89.6%)
Drowsiness	141 (73.1%)
Feeling in a “fog”	184 (95.3%)
Feeling slowed down	154 (79.8%)
Difficulty concentrating	178 (92.2%)
Dizziness	183 (94.8%)

## Discussion

This is the first study to investigate concussion knowledge, attitudes, and reporting intention in full-contact combat sport athletes who are exposed to a very high risk of concussion. It was revealed that competitive Muay Thai kickboxing athletes have reasonably good concussion knowledge, attitudes, and reporting intention, although there is still room for further improvement.

The mean CKI score in competitive Muay Thai kickboxing athletes was 78%. This level of concussion knowledge is very similar to that observed among athletes in intercollegiate sports (i.e. American football, soccer, volleyball, water polo), who had a mean CKI score of 77% (Chinn & Porter, 2016). It is also similar to the level of concussion knowledge found among parents or primary caregivers of children presenting to hospital for evaluation of musculoskeletal injury or concussion, who had a mean CKI score of 74% (Lin et al., 2015).

The competitive Muay Thai kickboxing athletes in this study displayed very good concussion attitudes, with a mean CAI score of 84%. This is almost identical to the concussion attitudes among parents or primary caregivers of children presenting to hospital for evaluation of musculoskeletal injury or concussion, who were reported to have a mean CAI of 84% (Lin et al., 2015). In contrast, the mean CAI score for intercollegiate sports athletes was observed to be only 63% (Chinn & Porter, 2016).

In regard to concussion reporting intention, 69% of the competitive Muay Thai kickboxing athletes in this study indicated that they were likely to report concussion symptoms. Unfortunately, there is scant literature available for direct comparisons, thereby making it difficult gauge whether kickboxing athlete are more or less likely to report concussion symptoms relative to athletes in other sports. Kroshus and colleagues (Kroshus et al., 2014) used the same measure of concussion reporting intention in a sample of male ice hockey players from six collegiate teams; however, they did not report the proportion of athletes indicating that they were likely to report concussion symptoms. Rather, they reported the six teams’ mean scores to range between 2.00 and 3.38, where lower scores indicated better concussion reporting intention. In the present study the mean score was 2.15 (*SD* 1.16), suggesting that kickboxing athletes have, on average, similar or slightly better concussion symptoms reporting intention than ice hockey players.

It is unclear whence the kickboxing athletes in the present study have obtained their concussion knowledge and attitudes, and future studies are encouraged to explore where and how these athletes acquire concussion information. Because there currently is no mandated concussion education program for these athletes, it is reasonable to believe knowledge is derived from a variety of sources, including but not necessarily limited to: word of mouth (e.g. other athletes, coaches, friends, or family), news media (e.g. print and online newspapers, television, and radio), popular entertainment media (e.g. television, magazines, and movies), formal education (e.g. athletic training, strength and conditioning, and medical and health sciences), and miscellaneous Internet sources (e.g. blogs, podcasts, combat sport websites, medical and health websites). It is expected that the accuracy and currency of the concussion information vary considerably across the various information sources. Consequently, kickboxing athletes may be vulnerable to the influence of misleading concussion information. It is therefore recommended that all full-contact kickboxing athletes and coaches regularly undertake concussion education programs based on current best evidence. This recommendation is also in line with the current position statement on concussion in combat sports by the Association of Ringside Physicians (Neidecker et al., 2017). Ideally, such concussion education programs would be mandated by relevant sport governing bodies and sanctioning authorities to ensure maximum uptake and compliance.

The present study did not reveal any specific predictors of better concussion knowledge, attitudes, or reporting intention, which suggest that generic concussion education programs may be appropriate for kickboxing athletes. However, future studies should be adequately powered to explore a wider range of potential predictors, and if any are revealed, these may in turn be used to tailor concussion education programs for specific subgroups of kickboxing athletes.

The present study revealed some specific concussion knowledge gaps among this cohort of kickboxing athletes. For instance, the majority of athletes failed to understand the limitations of brain imaging in concussion evaluation, and to distinguish between loss of consciousness and coma. Such specific concussion knowledge gaps could be addressed by concussion education programs targeted towards kickboxing athletes. It is vital that concussion education programs are based on psychosocial theoretical frameworks and best practice to optimize knowledge translation (Register-Mihalik et al., 2017; Mrazik et al., 2015). However, it is worth pointing out that the findings of the present study suggest that there is room for only modest overall improvements in concussion knowledge, attitudes, and reporting intention. This, in turn, suggests that the return on investment of implementing concussion education programs in terms of reducing the burden of concussion in kickboxing may be limited. Future studies are therefore encouraged to examine the effectiveness and cost-effectiveness of implementing concussion education programs for kickboxing.

The main limitations of this study pertain to the study sample. Firstly, participants volunteered to participate in this study, and it is possible that the opinions and beliefs of those who chose to participate were not necessarily representative of the population of Muay Thai kickboxing athletes from which the sample was derived (selection bias). In addition, the generalizability of the findings herein may be limited due to the sampling being restricted to Muay Thai kickboxing athletes in North America (i.e. the United States of America and Canada). It is possible that the concussion knowledge, attitudes, and reporting intentions of the Muay Thai kickboxing athletes in this study differ from those in other parts of the world. Moreover, further research is needed to investigate whether the situation in other full-contact combat sport differs from that reported in the present study of Muay Thai kickboxing athletes.

## Conclusions

Competitive Muay Thai kickboxing athletes appear to have reasonably good concussion knowledge, attitudes, and reporting intention; however, there is still room for further improvement. It is recommended that concussion education programs based on current best evidence are made available for all full-contact kickboxing athletes and coaches.
